# Does osteoarthritis physiotherapy research in South Korea align with the National Institute for Health and Care Excellence guidelines: a systematic review of English and Korean literature

**DOI:** 10.1186/s41927-025-00496-w

**Published:** 2025-05-27

**Authors:** Mi La Park, Nico Magni, Daniel W. O’Brien

**Affiliations:** 1 Department of Physiotherapy, School of Clinical Sciences, Auckland University of Technology, Auckland, New Zealand; 2 Active Living and Rehabilitation: Aotearoa New Zealand, Pain and Musculoskeletal Conditions Research Group, Health and Rehabilitation Research Institute, School of Clinical Sciences, Auckland University of Technology, Auckland, New Zealand

**Keywords:** Culture, Guidelines, Osteoarthritis, South Korea

## Abstract

**Background:**

Osteoarthritis (OA) is a leading cause of lower limb disability worldwide, imposing significant socioeconomic and personal burden. Thus, many internationally recognised organisations have developed management guidelines for this condition. Among these, the National Institute for Health and Care Excellence (NICE) recommends four first-line approaches to osteoarthritis management: education, exercise, self-management, and weight management. Despite the development of guidelines, adherence to OA management recommendations appears to be suboptimal internationally, and little is known about guideline adherence in South Korea. This study aimed to explore whether research-based physiotherapy interventions for OA in South Korea align with the NICE guidelines.

**Methods:**

A comprehensive search was conducted across multiple Korean and English electronic databases, including the Korea Citation Index (KCI), Korean Studies Information Service System (KISS), MEDLINE, EMBASE, CINAHL, SPORTDiscus SCOPUS, and Google Scholar. Twelve randomized controlled trials conducted in South Korea met the inclusion criteria, with sample sizes ranging from 20 to 60 participants. Participants’ mean age ranged from 57 to 75 years, and their Body Mass Index (BMI) varied from 23.00 to 25.68 kg/m². The primary outcome measure was the alignment of interventions with NICE OA guidelines, assessed using a scoring system (0–2 points per study) developed specifically for this review. Additionally, the methodological quality of included studies was evaluated using the Physiotherapy Evidence Database (PEDro) scale.

**Results:**

Most studies had poor methodological quality (PEdro scale range: 3–5). Only 42% of the Korean studies aligned with the NICE OA recommendations. Commonly applied interventions were predominantly passive, such as heat therapy, electrotherapy, and kinesiology taping, none of which are recommended by NICE.

**Conclusions:**

A discrepancy was found between research-based physiotherapy interventions for osteoarthritis in South Korea and the therapeutic approaches recommended by the National Institute for Health and Care Excellence guidelines. Factors such as a lack of evidence-based education, research, healthcare funding in South Korea, and cultural health experiences and expectations of the patients may have contributed to these findings. These results could help develop new strategies for improving osteoarthritis management in South Korea.

**Supplementary information:**

The online version contains supplementary material available at 10.1186/s41927-025-00496-w.

## Background

Osteoarthritis (OA) is a rapidly growing public health concern affecting 7% (500 million) of the global population [1–3]. Its international prevalence increased by 48% between 1990 and 2019, and in 2019, it was identified as a leading cause of lower limb disability [1, 2, 4]. OA can limit the ability of an individual to engage in activities due to pain, weakness, and joint stiffness [5]. These symptoms negatively affect emotional well-being and social life [6, 7]. Nearly 40% of OA patients experience anxiety and depression [8]. The interaction between physical limitations and depressive symptoms can prevent individuals from engaging in work or other activities [8, 9].

According to Arthritis New Zealand [10], in 2018, the direct and indirect costs for OA management were $12.2 billion, including $7.9 million in lost well-being, $3.3 billion in lost productivity, and $993 million in healthcare costs. In the USA, direct medical expenses for OA were approximately $72 billion between 2008 and 2011, and more recent statistics showed that OA-related costs have increased annually by 5.7% from 1996 to 2016 [11, 12]. Globally, the average annual direct cost for OA care ranges from $300 to $17,700, with an average cost per patient of $6,300 [12]. These statistics indicate that OA places an increasing financial burden on global healthcare systems [5, 12, 13]. Similar trends have shown an increase in the prevalence of OA and related healthcare expenditures in South Korea [2, 14], and the incidence of symptomatic OA is expected to increase with the predicted growth in the number of people in the country aged > 65 from 15.6% in 2022 to 38.2% by 2050 [15]. In 2020, the combined costs of outpatient and inpatient care for OA in South Korea reached $1.14 billion [16].

OA’s escalating socioeconomic and personal burden has prompted researchers and clinicians to develop efficient management strategies and enhanced care protocols for individuals with this condition [17, 18]. Several internationally recognised organizations, such as the American College of Rheumatology (ACR), Osteoarthritis Research Society International (OARSI), and the National Institute for Health and Care Excellence (NICE), have issued clinical practice guidelines (CPGs) for OA management [18–20]. As first-line treatments for OA, these guidelines consistently recommend self-management strategies-such as exercise, weight management, and patient education-to reduce pain, improve function, and enhance quality of life [4, 8, 19–21]. Because OA is a chronic disease, these approaches empower individuals to take an active role in symptom management by integrating education that fosters behavioural change and encourages active coping strategies [18, 19]. When first-line treatments are insufficient, pharmacological interventions, such as nonsteroidal anti-inflammatory drugs (NSAIDs), are recommended as second-line options, while surgical procedures are reserved for cases unresponsive to conservative interventions [19].

Although these CPGs have been developed and are widely available, their implementation remains suboptimal, with inconsistent adherence to OA management recommendations [3]. Many healthcare providers continue delivering low-value care with minimal patient benefits [13, 18, 22]. Research indicates that a similar pattern of low-value care has been observed in South Korea, where non-surgical interventions for OA frequently involve the use of electrophysical modalities (e.g., superficial heat, transcutaneous electrical nerve stimulation [TENS], interferential current therapy [ICT], laser therapy, and ultrasound) [23, 24]. A scoping review by Diarbakerli, Thoreson [25] on musculoskeletal clinical research in Sweden revealed that delivering low-value care and replicating interventions in research could be attributed to insufficient funding or a general scarcity of local research. This determination may also apply to South Korea, where only low-value treatments, such as heat/cold therapy, ICT, and ultrasound, are funded by the National Insurance system (NIS) [24]. Presently, limited studies have been conducted on the physiotherapy management of patients with OA in South Korea, and their findings highlight possible issues with applying evidence-based practices (EBP) [26]. The prevalence of these treatments and their replication in research likely reflects common practices in routine treatment in South Korea.

The aim of the systematic review is to explore physiotherapy-based OA research conducted in South Korea and its alignment with the international (NICE) OA guidelines [19]. By determining whether a gap exists between recommended practices and physiotherapy-based OA research in South Korea, this study may encourage future adherence to the NICE guidelines. In addition, exploring South Korean cultural, social, and educational factors may enable the identification of aspects of CPGs that require improvement for future implementation.

## Methods

### Terms and search strategies

This study was registered in PROSPERO (ID: CRD42023452416). Electronic databases were searched from July 2023 to August 2023 to identify clinical trials in English and Korean literature focusing on physiotherapy-based OA management in South Korea. Two Korean research databases, the Korea Citation Index (KCI) and the Korean Studies Information Service System (KISS), were used to access Korean articles. English literature searches were performed using MEDLINE, EMBASE, CINAHL, SPORTDiscus (accessed via the EBSCO health databases), SCOPUS, and Google Scholar. The primary search used three English and Korean terms tailored to specific databases. The first English language search terms, reported under the heading “Search 1” (S1), focused on osteoarthritis. The Medical Subject Headings (MeSH) term “osteoarthritis” [MeSH] was used along with its alternative terms. “Search 2” (S2) targeted physiotherapy and included the MeSH term “rehabilitation” [MeSH]. “Search 3” (S3) focused on “South Korea”, using the MeSH term “Republic of Korea” alongside relevant alternative terms. Table 1 outlines the search terms, their alternatives in English and Korean, and their truncated symbols. An example of the search strategy and results for the EBSCO Health Database and KCI is presented in Appendix 1.

**Table 1:** English and Korean search terms and their alternatives

Order of terms searched	English	Korean
Search 1 (S1)	(osteoarthritis OR osteoarthrit* OR “degenerative arthriti*” OR arthrosis)	(골관절염 OR “퇴행성 관절염” OR 관절증)
AND		
Search 2 (S2)	(physiotherp* OR “physical therap*” OR rehabilitation OR treatmen* OR “conservative manageme*” OR therapy)	(물리치료 OR 재활 OR 치료 OR “보존적 치료”)
AND		
Search 3 (S3)	(“South Korea” OR “Republic of Korea” OR Korean OR “Korean patient*”)	(한국 OR 한국인 OR “한국 환자”)

### Eligibility criteria

The eligibility for this review was limited to randomised controlled trials conducted by physiotherapists in South Korea. Studies had to include participants aged ≥ 50 years with a clinical diagnosis and clinical symptoms of OA related to weight-bearing joints. There were no restrictions on the grade or stage of OA. Studies were included if published after 2010 because they had a greater chance of aligning with the current NICE guidelines and reflecting current clinical practice. However, studies were excluded if they included participants who applied the intervention themselves, were aged < 50 years, presented with rheumatoid arthritis, or were undergoing post-surgical physiotherapy.

### Data extraction

The primary researcher (MLP) extracted the characteristics of each study to facilitate the comparisons. Data were grouped under the following headings: author, mean age, sample size, body mass index (BMI), physical activity (PA), selection criteria, interventions, key findings, and alignment with the NICE guidelines. When the mean participant BMI was not provided, the primary researcher calculated it using the available mean height and weight. It is important to note that BMI categories differ between Korean and Western populations [16, 27], as outlined in Table 2. (All references to BMI in this manuscript use Korean categories.)

**Table 2:** Body mass index categories for Korean and Western populations

Population	Underweight	Normal weight	Overweight	Obesity
Korean	<18.5 kg/m²	18.5–22.9 kg/m²	23–24.9 kg/m²	≥ 25 kg/m²
Western	<18.5 kg/m²	18.5–24.9 kg/m²	25–29.9 kg/m²	≥ 30 kg/m²

### Alignment with the NICE guidelines

Currently, there is no dedicated tool to assess the extent to which research aligns with international guidelines on OA management. Thus, the primary researcher developed a scoring system to assess and measure the alignment. Table 3 summarises the recommendations from the NICE guidelines and the scoring points of the primary reviewer for conservative OA management. The guidance provided by the NICE was selected for its high-quality appraisal, comprehensive scope, and strong editorial independence, making it a reliable reference for this review [28].

**Table 3:** National Institute for Health and Care Excellence guidelines for osteoarthritis interventions and scoring points

Recommended (2 points)	To be considered (1 point)	Not recommended (0 points)
Therapeutic exercise – Local muscle strengthening – General aerobic fitness Weight management Education Self-management	Manual therapy – Only hip or knee – Combine with therapeutic exercise Walking aids	Electrotherapy – TENS – U/S – Interferential – Laser – Pulsed short-wave therapy – Extracorporeal shockwave therapy

Under the scoring system developed by the primary researcher, interventions were given 2 points if they included a recommended core intervention that aligned with the NICE guidelines, 1 point if they included a recommended intervention which is considered within the guidelines but is not considered a core recommendation, and 0 points if they did not include a recommended intervention. Table 3 summarises the recommendations from the NICE guidelines [19] and the scoring points of the student reviewer on conservative OA management.

### Methodological quality

The Physiotherapy Evidence Database (PEDro) scale (Table 4) was used to analyse the methodological quality of the included studies. It is widely used and accepted as a comprehensive tool for evaluating the methodological quality of physiotherapy trials [29]. This checklist consisted of 11 items designed to assess a study’s internal and external validity. The scoring system of the PEDro scale operates by awarding 1 point for each “yes” and 0 points for each “no.” Because Item 1 was not included in calculating the total score as it represents external validity, a total appraisal score out of a maximum of 10 was used to indicate the overall quality of each reviewed study. Studies scoring < five were classified as “low quality,” those scoring six to eight as “moderate quality,” and those scoring ≥ nine as “high quality.”

**Table 4:** Quality assessment of the selected studies with Physiotherapy Evidence Database scale

Scores for Physiotherapy Evidence Database criteria														
	1*	2	3	4	5	6	7	8	9	10	11	QS	Methodological Quality	IVS
Cho et al. (2015)	Y	Y	Y	Y	N	Y	Y	N	N	Y	Y	7/10	Moderate	4
Choi et al. (2015)	Y	Y	N	Y	Y	N	N	N	N	Y	Y	5/10	Low	2
Chung and Cho (2015)	Y	Y	N	Y	N	N	N	N	Y	Y	Y	5/10	Low	1
Jeon et al. (2012)	Y	Y	N	Y	N	N	N	N	Y	Y	Y	5/10	Low	2
Kim et al. (2020)	Y	N	N	Y	N	N	N	N	Y	Y	Y	4/10	Low	1
Kim and Yu (2020)	Y	Y	N	Y	N	N	N	N	Y	Y	Y	5/10	Low	2
Lee et al. (2017)	Y	N	N	Y	N	N	N	N	N	Y	Y	3/10	Low	0
Lee et al. (2016)	Y	N	N	Y	N	N	N	N	N	Y	Y	3/10	Low	0
Lee et al. (2015)	Y	Y	N	N	N	N	N	N	Y	Y	Y	4/10	Low	2
Lee et al. (2012)	Y	N	N	Y	N	N	N	N	Y	Y	Y	4/10	Low	1
Oh et al. (2020)	Y	N	N	Y	N	N	N	N	N	Y	Y	3/10	Low	0
Park and Kim (2017)	Y	Y	N	Y	N	N	N	N	Y	Y	Y	5/10	Low	1

* Criteria do not contribute to the total score; (n) Criteria used to obtain an IVS; IVS, internal validity score; N, no; Y, yes; QS, quality score

In this review, the internal validity of each study was evaluated by calculating the internal validity score (IVS), an approach used in other systematic reviews [30, 31]. According to Bassett, Lingman [30], PEDro items 2, 3, 5, 6, 7, 8, and 9 represent the elements of internal validity, and the points awarded to each of these items can be used to determine an IVS. Studies achieving a final IVS of six or seven are categorised as having “high” methodological quality, an IVS of four or five indicates “moderate” methodological quality, and an IVS of less than three suggests “limited” methodological quality [30, 31].

### Cross-assessment of studies in Korean and English literature

This systematic review involved a cross-assessment of studies published in Korean and English. This procedure was performed by two bilingual individuals (MLP, SR), each with a comprehensive understanding of the Korean and English languages and a background in physiotherapy. The aforementioned evaluation of the quality of English and Korean studies was performed in two steps. The primary researcher independently critiqued the studies using two quality tests. Next, another research team member (NM) appraised all articles in English, and a second reviewer (SR: a bilingual physiotherapist) conducted the same quality assessment of all articles written in Korean. When disagreements occurred, an additional team member (DOB) was asked to moderate until a consensus was reached. Cohen’s kappa was used to assess the level of agreement among the reviewers when scoring the research papers. The calculated kappa statistic provides a quantitative measure of inter-rater reliability, helping to gauge the consistency of assessments beyond what would be expected by chance alone [32].

## Results

### Search results and study selection

**Fig. 1:**
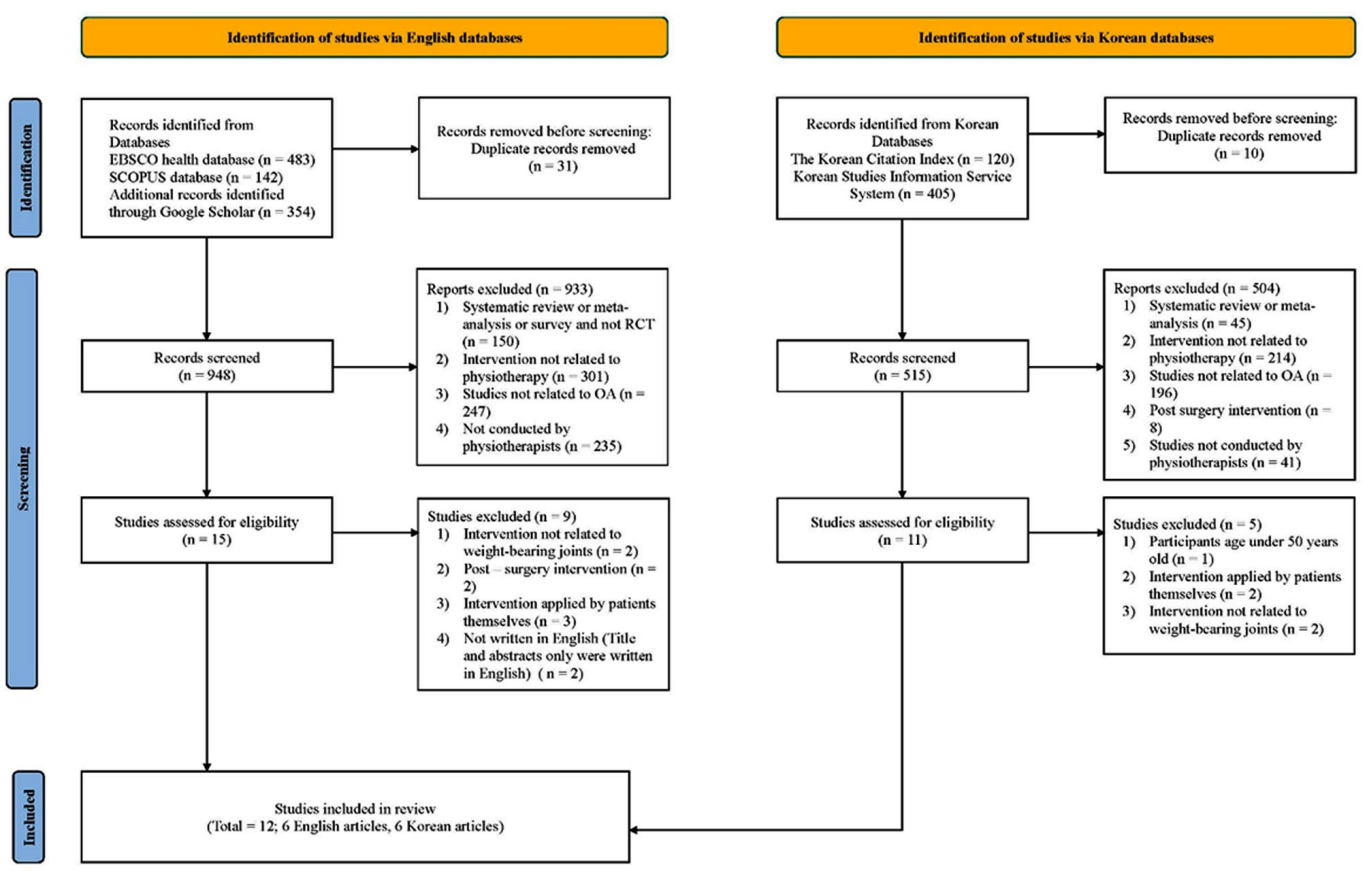
Identification of studies via databases and registers

The PRISMA flow diagram is shown in Fig. 1. The initial search identified 1,504 articles (English articles, 979; Korean articles, 525). Duplicates (n = 41) were removed, and the titles and abstracts of the remaining articles were screened. Following this screening, 26 studies were subjected to a full-text review. Of these, nine English studies were excluded because two did not address symptoms and diagnoses associated with weight-bearing joints, two addressed the subject of post-surgery management, three addressed interventions applied by patients, and two had only titles and abstracts written in English. Of the Korean studies that underwent a full-text review, five were excluded. The reasons for the exclusion were similar to those indicated in the English studies. In addition, one Korean study included participants aged < 50, which was an exclusion criterion. Twelve articles (six in Korean and six in English) were included for quality appraisal and data extraction. No additional articles were identified after screening the reference lists.

### Participants

As shown in Table 5, the sample size, participant demographic characteristics, and inclusion and exclusion criteria varied across studies. The sample size ranged from 20 to 60 participants, and the mean participant age ranged from 57 to 75 years. None of the selected English and Korean studies provided the participants’ BMI scores, so the primary researcher calculated these for all but one of the studies using demographic data. Because the study by Jeon, Kwon [33] did not report details of the participants, the average BMI for this population could not be calculated. In the remaining 11 studies, the average BMI in each group ranged from 23.00 to 25.68 kg/m². Four studies [34–37] revealed that their participants were classified as obese (≥ 25 kg/m²). Of the participants across all studies for which calculations could be made, approximately 25% were on the border between overweight and obese. Furthermore, one-third of the participants across all studies (98 out of 327) had a BMI that was marginally above the normal range (≥ 22. kg/m²). None of the included studies reported the participants’ baseline physical activity levels.

**Table 5:** Study characteristics of reviewed literature

Author(s) (Year)	Participants				Selection criteria		Intervention		Key findings		Alignment with NICE guidelines
	Numbers (*N*) Mean Age	BMI EG CG		PA	Inclusion	Exclusion	EG	CG	EG	CG	
Cho et al. (2015)	*n* = 46 EG: *n* = 23 Age = 58.2$\pm$4.5 CG: *n* = 23 Age = 57.5$\pm$4.4	25.32	25.31	N/R	Age: > 50, symptom ≥ 1 year, radiographic confirm, pain: > 50/100 walking, no ligament injury	Fracture, surgery, neurological disorder/vestibular problem, taking medication	KT	Placebo KT	Improved VAS ROM proprioception	No improvement: VAS ROM proprioception	0
Choi et al. (2015)	*n* = 30 EG 1: *n* = 10 EG 2: *n* = 10 CG: *n* = 10 EG 1, 2 & CG Mean age = 72.8$\pm$8.8	Mean EG & CG = 24.3$\pm$2.5	MeanEG & CG = 24.3$\pm$2.5	N/R	Radiographic confirm	Knee surgery: > 6 month, BMI: < 38, steroid injection, RA	EG 1: EMGBF -20 min -3 times/week -8 weeks EG 2: USBF -same as EG 1	HP: 15 min U/S: 5 min TENS: 15 min -3 times/week -8 weeks	EG 1 & 2 Improved VAS & strength	No improvement: VAS & strength	2
Chung and Cho (2015)	*n* = 30 EG: *n* = 15 Age = 65.0$\pm$2.07 CG: *n* = 15 Age = 62.4.5$\pm$2.27	23	23.08	N/R	OA confirms by doctor	No disorder for communication	Micro-current, HP, U/S & TENS -3 times/week -6 weeks	HP, U/S & TENS -3 times/week -6 weeks	Improved VAS MPQ TUG Weight distribution	Improved VAS MPQ TUG Weight distribution	0
Jeon et al. (2012)	*n* = 60 EG1: *n* = 20 EG2: *n* = 20 EG3: *n* = 20 No mention of participants characteristics No control group			N/R	Age: > 65–85, morning stiffness: < 30 min, crepitus & tenderness in knee, limited ROM, pain in rest	RA, TKJR, secondary to OA from trauma, communication deficit, knee treatment: < 1 month	Group 1: U/S -5 min - Not mention frequency -6 weeks Group 2: Laser -5 min -6 weeks Group 3: Combining US-laser - 5 min for laser -6 weeks		Group 1 - No VAS & PPT improved Group 2 & 3 - VAS & PPT improved		0
Kim et al. (2020)	*n* = 30 EG: *n* = 15 Age = 69.3$\pm$4.2 CG: *n* = 15 Age = 70$\pm$5.3	23.73	23.55	N/R	Age: > 65, K–L: 1-II	Neurological, cardiovascular disease & RA	Resistance exercise with band + HP, U/S & interference wave -2 sets/10 reps -3 times/week -4 weeks	HP, U/S & interference wave	Improved VAS & K-WOMAC	Improved VAS & K-WOMAC	2
Kim and Yu (2020)	*n* = 30 EG: *n* = 15 Age = 70.40$\pm$3.11 CG:*n* = 15 Age = 69.8$\pm$4.52	24.67	23.82	N/R	Age: > 65, OA confirms by doctor & radiographic, WOMAC (Pain): < 15 points, WOMAC (function): < 51 points, knee flexion: > 90 degree	THR, TKJR, Severe neurological, cardiovascular disease, communication deficit	Sensory motor training by using sling -40 min -3 times/week -6 weeks	HP (20 min), U/S (5 min) & interference wave (15 min) -3 times/week -6 weeks	Improved WOMAC function, No improved pain	No improvement	0
Lee et al. (2017)	*n* = 20 EG: *n* = 10 Age = 64.2$\pm$4.1 CG: *n* = 10 Age = 67.2$\pm$5.9	23.82	25.04	N/R	Age: > 55–70, K–L > 2	Neurological or cardiovascular disease, RA & surgery	ESWT -4 Hz &1,000 times using focus-type head Combining with HP (20 min), U/S (5 min) & interference current therapy (15 min) -3 times/week -4 weeks	HP (20 min), U/S(5 min) & interference current therapy (15 min) - 3times/week - 4 weeks	Improved VAS & K-WOMAC significantly	Improved VAS & K-WOMAC	0
Lee et al. (2016)	*n* = 30 EG: *n* = 15 Age = 72.0$\pm$4.9 CG: *n* = 15 Age = 73.1$\pm$5.8	25.60	24.57	N/R	Age: > 55–70, K–L: > 2	Fracture & ligament injury, no other soft tissue injury, no dysesthesias	KT, HP & interference wave -3 times/week -4 weeks	HP & interference wave -3 times/ week -4 weeks	Improved VAS, K-WOMAC & ROM	Improved VAS & K-WOMAC	0
Lee et al. (2015)	*n* = 30 EG: *n* = 15 Age = 57.25$\pm$7.59 CG: *n* = 15 Age = 61.82$\pm$8.07	24.65	24.83	N/R	Not mentioned	Lower limb surgery, cortisone injections & inflammatory medications: ≤ 3-month, ligament injury, inflammatory disease, no independent gait	Manual joint mobilisation + HP (20 min), U/S (5 min) & TENS (20 min) Manual therapy -3 times/week -18 min -4 weeks	HP (20 min), U/S (5 min) & TENS (20 min) -3times/week -45 min -4 weeks			0
Lee et al. (2012)	*n* = 23 EG: *n* = 12 Age = 74.92$\pm$5.16 CG: *n* = 11 Age = 74.18$\pm$4.55	24.65	24.62	N/R	Diagnosed with OA, ongoing treatment with HP and other electric modality	Previous knee or back surgery, neurological disorder, RA, taking pain medication, hypertension, severe inflammation or swelling	Complex exercise program + HP & interference wave) 40 minutes 3 times/week 10 weeks	HP & interference wave 40 minutes 3 times/week 10 weeks	Improved VAS and muscle activation	Improved VAS and muscle activation	2
Oh et al. (2020)	*n* = 26 EG: *n* = 13 Age = 64.5$\pm$3.6 CG: *n* = 13 Age = 67.2$\pm$5.3	23.58	24.43	N/R	Age: > 60, OA	Neurological diseases & surgery	Visual feedback balance training + HP & wet therapy, ICT, U/S -3 times/week -8 weeks	Muscle strengthening exercise with elastic band + HP & wet therapy, ICT, and U/S -3 sets/10 reps Knee extension -3 times/ week - 8 weeks	Both groups improved VAS & K-WOMAC		2
Park and Kim (2017)	*n* = 25 EG *n* = 12 Age = 75.42$\pm$7.87 CG *n* = 13 Age = 68.85$\pm$3.56	25.68	24.92	N/R	Diagnosed by orthopaedic doctor, K–L: I–III, knee symptoms: > 3 months	Vestibula disease, vision deficits, using walking aids, VAS: 4–7/10	Lower limb strengthening exercise (15 min) + balance exercise (15 min) + HP, Interferential current & aerobic exercise (bike & treadmill) -3times/week -4 weeks	Lower limb strengthening exercise (30 min) + HP (15 min), Interferential current (15 min) & aerobic exercise (bike & treadmill) -3times/week - 4 weeks	Both groups improved WOMAC, NRS, ROM, CST& BBS		2

While all participants in the 12 studies were diagnosed with OA, three studies [33, 38, 39] did not clearly specify the diagnostic criteria. In most studies, researchers stated that orthopaedic specialists or general practitioners confirmed the diagnosed OA based on radiographic findings. In five studies, the Kellgren–Lawrence (K–L) classification system was used as an inclusion criterion for participation [35–37, 40, 41], with variations in the K–L grades (I–III) utilized among these studies. Other studies did not employ any other criteria beyond OA diagnosis for inclusion. However, Jeon, Kwon [33] did not provide the inclusion criteria for their study. Studies commonly reported the following exclusion criteria: surgery, neurological disorder, and rheumatoid arthritis.

### Intervention and control

The reviewed interventions included kinesiology taping (KT), electrical modalities, exercise-based treatments, and manual joint mobilisation, often combined with electrotherapy. Cho, Kim [34] and Lee, Yi [36] investigated the efficacy of KT in managing knee OA. Additionally, three trials [33, 35, 38] were conducted to test the effect of various electric modalities, including microcurrent, laser, and extracorporeal shock waves, on the symptoms of patients with knee OA. Half of the included studies focused on exercise-based interventions for muscle strengthening and balance [37, 41–44]. Oh, Lee [43] compared visual feedback balance exercises with muscle strengthening exercises. For their research, the experimental group used visual feedback balance exercises, while the control group engaged in localized muscle strengthening exercises; the NICE guidelines do not recommend the balance exercises used in the experimental group. Similarly, Oh, Lee [43], Kim and Yu [44] explored the impact of non-recommended treatment by using sensorimotor (balance) exercises as the primary intervention, which also diverges from the NICE guidelines. Lee, Kwon [39] examined the efficacy of manual joint mobilization combined with electrical modalities rather than exercise.

Notably, four of the five exercise-based studies incorporated a combination of electrotherapy (heat packs, interferential current waves [ICT], and ultrasound [U/S]) as part of their primary interventions. Table 5 shows that ten studies used similar modalities, such as heat packs, ICT, and U/S, as interventions for the control groups. Passive electrotherapy was consistently applied to the experimental and control groups. The frequency of intervention for most studies was three times per week, and the treatment period ranged from 4 to 12 weeks (Table 5).

### Methodological quality

The methodological quality of the studies was assessed using the PEDro scale and an IVS. Table 5 shows (see Table 5 at the end of the manuscript) that the overall quality of the studies was predominantly low, with 11 studies scoring 3 to 5 out of 10 on the PEDro scale. One study conducted by Choi, Kim [40] scored 7 out of 10 on the PEDro scale, indicating moderate quality. None of the reviewed studies was of high quality.

Regarding IVS, four of the 12 reviewed studies received scores of 0, and six scored 1 or 2. Choi, Kim [40] demonstrated moderate quality, scoring an IVS of 4, matching moderate standing on the PEDro scale. None of the 12 studies met the criteria for item 8, which was related to group dropouts. Additionally, 11 of the 12 studies did not satisfy items 5, 6, and 7, demonstrating the study’s internal validity through the extent to which the participants, therapists, and assessors were blinded.

### Inter-rater reliability of cross-assessment of studies

The inter-rater reliability between the reviewers of English studies (MLP, NM) was moderate to substantial, with a Cohen’s kappa coefficient of 0.61, indicating good agreement beyond what would be expected by chance alone. The inter-rater reliability between the reviewers of the Korean studies (MLP and SR) was substantial, with a Cohen’s kappa coefficient of 0.67, indicating strong agreement beyond what would be expected by chance alone.

### Alignment with NICE guidelines

Regarding the alignment between the interventions and the NICE OA guidelines, five reviewed studies scored 2 points, including the recommended intervention. Four of these studies used local muscle-strengthening exercises [37, 40, 41, 43], whereas the remaining study assessed the effects of whole-body strengthening exercises [42]. Notably, four of the studies that scored 2 points combined electrical modality treatments and exercise [37, 41–43], and only one study [40] exclusively focused on the effects of patients engaging in targeted strengthening exercises.

Seven studies scored 0 points because they included treatment interventions that did not align with the NICE guidelines. Two of these studies employed KT [34, 36] and three utilised electric modalities such as micro-current wave, U/S, laser, and extracorporeal shockwave therapy [33, 35, 38]. Although Lee, Kwon [39] applied manual therapy, a recommended intervention, they received a score of zero because they combined it with a passive form of electrotherapy instead of combining it with exercise. Another study [44] implemented balance exercises that did not adhere to the NICE guidelines, which recommend local muscle strengthening and aerobic exercises.

The control groups in all 12 studies received interventions not recommended by the NICE guidelines, such as heat packs, U/S, TENS, or interference waves. Furthermore, no studies applied other core management strategies recommended by the NICE guidelines for OA, such as weight management, education, and self-management.

## Discussion

This systematic review assessed whether research-based physiotherapy interventions for OA in South Korea aligned with the NICE OA guidelines. It is the first study to evaluate guideline adherence in South Korea in this context and including English and Korean publications in the review greatly enhanced its scope. The findings revealed a notable disparity between the NICE OA guidelines and interventions used in Korean studies. Only 42% of the studies met the guideline recommendations. The reason for this observed low alignment with the recommended guidelines is likely multifactorial. Our discussion explores potential explanations for this disparity in the context of the current literature, with a particular focus on contextual factors, including the South Korean health system and cultural and population differences.

International guidelines for OA management have been developed to provide optimal and effective care for patients with OA. The NICE OA guidelines recommend that patients with OA receive four fundamental interventions: education, exercise, self-management, and weight management [8, 19, 21]. However, this review showed that only about one-third of the studies conducted in South Korea applied management approaches that aligned with this recommendation. Instead of applying approved interventions, studies commonly used thermal modalities and electrotherapy, including micro-current waves, U/S, laser, extracorporeal shock wave therapy, and KT [34, 36, 45–48]. These OA management approaches have been categorized as low-value care for OA management [21, 22].

Factors that might explain some of the observed discrepancies in OA management are the structure and the funding approach of the South Korean National NHI system, which provides coverage for most of the population [49]. This system covers physiotherapy-specific interventions not endorsed by the NICE guidelines, such as thermal therapy (heat/cold), electrotherapy, and therapeutic exercises covered only by the South Korean NHI for postsurgical rehabilitation [24]. The interventions in these studies might mirror the routine treatment in South Korea, which is limited to modalities covered by the NHI system. This assessment is supported by Shim, Park [50], who highlighted that funding limitations resulted in > 90% of patients with OA in South Korea not receiving education on OA management or the importance of exercise. Similarly, Diarbakerli, Thoreson [25] contended that the common use of treatments not endorsed by international guidelines and the replication of such interventions in research could be linked to the insufficient funding of health schemes in which treatment is given and research is conducted.

The professional classification of physiotherapists in South Korea as medical technicians rather than as independent healthcare providers [51] may further restrict their abilities to implement treatments recommended by international guidelines but not covered by NHI. According to the World Confederation for Physical Therapy, 38% of the countries, including South Korea, have no autonomous practices for physiotherapists [52]. This lack of autonomy may restrict their abilities to fully implement non-insured, internationally recommended treatments for OA management.

Cultural contexts can influence approaches to healthcare delivery and research [53–55]. Most of the interventions used in the reviewed studies from South Korea used “passive” treatments applied “to” the participants. Sathiyamoorthy, Ali [53] undertook an extensive review of OA management across 75 Asian countries, including South Korea, and revealed that Asian patients often expect to rest and receive treatment from healthcare professionals rather than engage in active treatments. In Japanese patients, this tendency to expect passive treatment can be explained by the common belief that OA results from joint overuse [56]. Similarly, in South Korea, patients believe that OA is caused by joint overuse and the societal expectation that one engages in physically demanding occupations [55]. This belief explains why patients may play a more passive role in their rehabilitation, which could influence treatment options and be reflected in research settings [54]. Supporting this explanation, an Australian study by Naylor, Gibson [57] and a Canadian study by Tittlemier, Wittmeier [58] found that the expectations of patients, as well as the pursuit of financial returns by practitioners, caused some physiotherapists working in private musculoskeletal clinics to apply treatments not recommended by international OA guidelines. These findings suggest that patient expectations can influence practices utilized by physiotherapists. Therefore, patients’ cultural backgrounds and beliefs might explain the gap between physiotherapy research on OA management in South Korea and the current NICE OA guidelines. Therefore, for physiotherapists to transition from passive treatment, the government of South Korea may need to review the funding provided to the NHI system and the professional status of physiotherapists. Additionally, they should undertake widespread campaigns and educational initiatives to change the beliefs of the general population regarding OA and its treatment.

Western researchers and clinical groups designed most of the best-known international OA guidelines (NICE, OARSI, and ACR) for Western populations. This approach implies that inherent cultural biases may limit the transferability of recommendations to non-Western contexts. This research identified two examples where Western OA guidelines may not be as relevant to the South Korean population as their recommendations for weight management and physical activity.

Weight management is a first-line treatment in the NICE OA guidelines, as obesity is a high-risk factor for knee OA [19]. A high amount of body fat places a considerable load on the joints and contributes to metabolic factors that can cause systemic inflammation [16, 59]. Although the reviewed studies revealed that 25% of the participants were obese, most of these individuals barely met the South Korean threshold for obesity and were not considered obese in a Western context. Surprisingly, approximately 30% of the participants were only marginally above the normal-weight range. In contrast, most Western OA studies have reported much higher obesity rates among their participants [60]. While it remains legitimate to recognise obesity as a risk factor for OA in South Korea, the belief that prevails in Western countries that most patients with OA are obese may not hold true in South Korea. Further analysis by Kus, Yasaci [60] from the Our World in Data website indicated a significant increase in obesity rates in Western countries from approximately 10% to more than 30% between 1975 and 2016. In contrast, obesity rates in Southeast Asia, including South Korea, rose from approximately 0.5% to 4.5% over the same period [60]. This significant disparity between the populations of these two areas highlights the greater challenge obesity poses in Western societies, where weight management is emphasized as a crucial part of addressing OA over the long term, compared to Southeast Asian societies. Therefore, although weight management remains a valuable and beneficial intervention for all patients with OA, it may not be necessary to prioritize this intervention in South Korea. However, it has been reported that over the past 40 years, obesity prevalence has tripled in Western countries and increased by almost tenfold in Asian countries. Thus, weight management may become more important for OA treatment in this region in the future.

In recent years, there has been increasing international emphasis on the role of physical activity and exercise in managing OA [61, 62]. This is reflected in the NICE OA guidelines, which advocate exercise as the core treatment for OA [19, 61]. Specifically, these guidelines strongly recommend exercises that focus on strengthening and aerobic exercises to reduce the pain experienced and improve patients’ overall quality of life with OA [7, 8, 61, 62]. However, none of the five reviewed studies that chose exercise as the primary intervention measured the baseline physical activity levels of the participants. Additionally, the two reviewed studies applied balance exercises that were not recommended by the guidelines.

In the United States, many adults within community-based cohorts identified as having or being at high risk of knee OA did not meet the 2018 physical activity guidelines, which recommend 150 min of moderate-to-vigorous physical activity per week [63]. Moreover, an analysis of the U.S. Health and Nutrition Examination Survey found that only 12.3% of people aged > 50 years adhered to physical activity guidelines [63]. In contrast, in South Korea, over half of the population aged ≥ 50 performed regular exercise that meets the guidelines [64]. Therefore, some people with knee OA in South Korea may already be sufficiently active and, as a result, do not need additional exercises as part of their OA management. This contention is supported by Shim, Park [50], who’s nationwide Korean study revealed that 39% of the people affected by knee OA were physically active, meeting the World Health Organization physical activity guidelines. Although exercise-based treatments are efficacious in Western countries, whether these approaches are effective in the South Korean context remains unknown.

As part of this review, methodological critique and an IVS assessment were conducted using the PEDro scale. Overall, the identified literature was predominantly of low quality, with most studies consistently not meeting the internal validity criteria. The failure of these studies may be intricately linked to the educational framework of South Korea. Research conducted by Lee, Oh [54] revealed that only 22% of therapists were capable of critically analysing the literature published in their field. This issue may be explained by the fact that only 33%–39% of physiotherapists are exposed to EBP and international guidelines during their training [26]. An additional gap in education was revealed by a comparative analysis of physiotherapy education and licensing examinations between the United States and South Korea by Kang, Lee [65], which revealed that key topics, such as safety considerations for assessment and treatment, professional competency, and research appraisal, were absent from the physiotherapy curriculum and licensing examinations in South Korea. A review using the PEDro scale found that most South Korean physiotherapy studies were of low quality, failing internal validity standards, likely due to educational gaps. Lee and Oh [66] found that only 22% of therapists could critically analyse literature, with just 33%-39% exposed to EBP and international guidelines during training [26]. Kang and Lee [65] highlighted missing topics in South Korea’s curriculum, including safety and research appraisal. In contrast, 56% of Canadian and 67% of U.S. physiotherapists receive training in critical research skills, underscoring the need for improved standards in South Korea [67].

Translating the international OA guidelines into Korean could help ensure that healthcare professionals can easily access and implement them in patient care. Language barriers often impede access to international OA guidelines. Michajlyszyn, Thompson [68] emphasised the importance of providing educational resources in the native languages of physiotherapy students to enhance their understanding and satisfaction. No comprehensive Korean translation of OA guidelines is currently available, and given that only selected sections are accessible to doctors, this does not help narrow the knowledge gap [16]. The identified low adherence to the NICE OA guidelines in South Korea may also be explained by the distinct healthcare funding system and cultural context of this country. The South Korean NHI primarily supports passive treatment for OA. Moreover, international guidelines developed in Western contexts may not be universally applicable, especially in countries such as South Korea, where the population differs from those in Western countries. Efforts are required to establish funding models in South Korea that support the delivery of high-value care and are responsive to cultural contexts. Bridging the knowledge gap in South Korea will involve enhancing physiotherapy education, reviewing funding criteria, translating full materials, and customising guidelines to reflect the unique cultural and healthcare context of the country.

The strengths of this systematic review is that it is the first bilingual study (Korean and English) to assess the alignment between physiotherapy-based OA research and best-practice clinical guidelines, that two physiotherapists/researchers critically reviewed all studies using a recognised appraisal tool, and the interventions used within the studies were scored against the internally regarded NICE guidelines [19]. The findings of this review provide insights into the conservative management of OA used in research in South Korea and contribute to the limited literature on research and adherence to guidelines in diverse regions. This study has some limitations. One limitation is that the results may not directly reflect routine clinical practice, which is a gap that often exists between theoretical research and practical applications. Therefore, caution must be exercised before assuming research trends reflect clinical practices. In addition, the absence of an accepted gold standard for assessing guideline adherence within research has led to the development of a customised scoring system for this review, which has not yet been validated. Finally, despite prior moderation, the difference in Cohen’s kappa values for inter-rater reliability between reviewers of the English (0.61) and Korean (0.67) studies suggests potential variations in interpretation or translation across studies.

Observational studies in South Korean clinical settings are needed to examine how theoretical guidelines are applied in practice and to identify barriers to their use. Surveys or qualitative research could further reveal physiotherapists’ current practices, challenges, and training needs in OA management. Future research should focus on translating and adapting international OA management guidelines for Korean and other Asian contexts, making them more accessible and relevant to local healthcare. Additionally, assessing patients’ baseline physical activity levels and treatment expectations will help create tailored, patient-centred interventions.

### Clinical implications

This review highlights a gap between physiotherapy research on OA treatment in South Korea and the NICE guidelines, with a prevalent reliance on passive treatments. Addressing this gap-where research is expected to reflect current clinical practices-requires a multifaceted approach. Integrating research principles, EBP, and guidelines adherence into the physiotherapy curriculum in South Korea could help bridge the gap. Furthermore, researchers should focus on conducting high-quality studies that align with the recommended interventions.

The identified non-adherence to the NICE OA guidelines in South Korea may also be explained by the distinct healthcare funding system and cultural context of this country. The South Korean National Health Insurance (NHI) primarily supports the provision of passive treatments for OA, limiting the implementation of active and evidence-based interventions. To align practice with international recommendations, healthcare funding system should support active interventions rather than passive treatments. Additionally, customizing guidelines to reflect the country’s unique cultural and healthcare context is essential.

By aligning education, policy, and clinical practice with evidence-based recommendations, South Korea can enhance OA management and improve patient outcomes.

## Conclusions

Despite the availability of numerous international clinical guidelines providing recommendations for the optimal management of osteoarthritis, this review found that the physiotherapy-based osteoarthritis research conducted in South Korea regarding the use of conservative management had low alignment with the recommendations of the well-respected and widely used National Institute for Health and Care Excellence guidelines. The specific characteristics of South Korean physiotherapy education, lack of accessibility to guidelines due to language issues, and the country’s healthcare systems and culture might contribute to these findings.

## Electronic supplementary material

Below is the link to the electronic supplementary material.

Supplementary Material 1

## Data Availability

Data supporting the findings of this review, including data collection forms, extracted data, and any additional materials used, are available upon reasonable request. For access, please contact the primary research (MLP) at parkmila@gamil.com.

## Abbreviations

ACR: American College of Rheumatology
BMI: Body mass index
CPGs: Clinical Practice Guidelines
EBP: Evidence-Based Practice
ESWT: Extracorporeal shock wave therapy
ICT: Interferential current therapy
IVS: Internal validity score
KIC: Korean Citation Index
KISS: Korean Studies Information Service System
K-L: Kellgren-Lawrence
NHI: National Health Insurance
NICE: National Institute for Health Care Excellence
OA: Osteoarthritis
OARSI: Osteoarthritis Research Society International
PA: Physical activity
PEDro: Physiotherapy Evidence Database
TENS: Transcutaneous electrical nerve stimulation
