# Supplementary material for: Does osteoarthritis physiotherapy research in South Korea align with the National Institute for Health and Care Excellence guidelines: a systematic review of English and Korean literature
Source: BMC Rheumatol. 2025 May 27;9:63. doi: 10.1186/s41927-025-00496-w (PMC12107800; doi:10.1186/s41927-025-00496-w)
Supplement: Supplementary file 1 — Supplementary Material 1 [file 41927_2025_496_MOESM1_ESM.docx]

# Appendices

## Appendix A

Database Search Strategy

Title

Does osteoarthritis physiotherapy research in South Korea align with the National Institute for Health and Care Excellence (NICE) guideline: A systematic review of English and Korean literature

Database collection

EBSCO health databases (Cumulative Index to Nursing and Allied Health Literature [CINAHL Complete], Medical Literature Analysis and Retrieval System Online [MEDLINE], SPORTDicus with Full Text)

(Search on 30/07/2023)

S1- (osteoarthritis OR osteoarthrit* OR "degenerative arthriti*" OR arthrosis)

Results- 185,274

Limited year from 2010 to 2023

Results- 123,653

S1 AND S2- (physiotherp* OR "physical therap*" OR rehabilitation OR treatmen* OR "conservative manageme*" OR therapy)

Results- 72,313

S1 AND S2 AND S3- ("South Korea" OR "Republic of Korea" OR Korean OR "Korean patient*")

Results- 1531

S1 AND S2 AND S3 NOT S4- ("surgical treatmen*" OR surgery OR injection OR medicat*)

Results- 489

Limited language: English

Results- 483

Korea Citation Index [KIC] databases

(Search on 18/08/2023)

S1- (관절염 OR “퇴행성 관절염” OR 관절증)

Results- 3,815

S1 AND S2- (물리치료 OR 재활)

Results- 1,106

S1 AND S2 AND S3- (한국 OR 한국인 OR “한국 환자”)

Results- 405

Languages: Korean

Limited to year: 2010-2023

Results- 240

S1 AND S2 AND S3 NOT S4- 수술 OR 약물OR 주사 OR “한방 치료”

Results- 120
